# Association Between Exposure Characteristics and the Risk for COVID-19 Infection Among Health Care Workers With and Without BNT162b2 Vaccination

**DOI:** 10.1001/jamanetworkopen.2021.25394

**Published:** 2021-09-01

**Authors:** Yonatan Oster, Shmuel Benenson, Limor Yochi Harpaz, Inon Buda, Ran Nir-Paz, Jacob Strahilevitz, Matan J. Cohen

**Affiliations:** 1 Faculty of Medicine, Hebrew University of Jerusalem, Jerusalem, Israel; 2 Department of Clinical Microbiology and Infectious Diseases, Hadassah Hebrew University Medical Center, Jerusalem, Israel; 3 General Management, Hadassah Hebrew University Medical Center, Jerusalem, Israel; 4 Clalit Health Services, Jerusalem District, Associated With the Hebrew University of Jerusalem, Israel

## Abstract

This case-control study examines the possible association between exposure characteristics and infection risk among vaccinated and nonvaccinated health care workers in Israel.

## Introduction

Mass vaccination of Israeli adults with the BNT162b2 vaccine has been associated with a substantially lower rate of SARS-CoV-2 infection.^1^ After the vaccination program, the Centers for Disease Control and Prevention eliminated the need for quarantine after exposure.^2^ However, there are still cases of SARS-CoV-2 infection among fully vaccinated individuals.^3,4^ We studied the possible association between exposure characteristics and infection risk among vaccinated and nonvaccinated health care workers (HCWs) in Israel.

## Methods

This case-control study was conducted between January 1 and March 31, 2021, at the Hadassah-Hebrew University Medical Center, a 2-campus medical center in Jerusalem, Israel. Since December 2020, the hospital’s management executed a vaccination program of HCWs with the BNT162b2 vaccine (Pfizer-BioNTech). Within 2 months, the program achieved almost complete vaccination coverage and a significant decrease in the number of infected HCWs.^4^ Of note, the dominant strain during the study period was the alpha variant.^5^

The Institutional Ethics Committee of Hadassah-Hebrew University Medical Center approved the study, with a waiver of informed consent owing to the noninterventional and retrospective nature of the study. This study was performed in accordance with the Strengthening the Reporting of Observational Studies in Epidemiology (STROBE) reporting guideline.

We compared all vaccinated HCWs who had positive test results for SARS-CoV-2 at least 2 weeks after receiving the second dose (vaccinated-positive) with the following 2 control groups: all nonvaccinated workers who had positive test results during the same period (nonvaccinated-positive) and a sample of vaccinated workers who were tested for SARS-CoV-2 but had negative results (vaccinated-negative) and were randomly selected from the laboratory database in a 1:3 ratio. Data were extracted from epidemiologic investigations, including demographic characteristics, symptoms, characteristics of exposure, and disease course. We compared the proportions of these characteristics with the χ^2^ test. Odds ratios (ORs) were calculated to compare the exposure of vaccinated-positive and nonvaccinated-positive HCWs with COVID-19–positive household members. A 2-sided *P* < .05 was considered statistically significant. Calculations were performed using WINPEPI, version 11.65 (Brixton Health).

## Results

A total of 171 HCWs were enrolled in the study. Of these, the mean (SD) age was 38 (13) years, 118 (69%) were female, and 7 (4%) were immunocompromised. There were 5312 vaccinated HCWs and 690 nonvaccinated HCWs as of March 31, 2021. Of the 5312 vaccinated HCWs, 27 (0.5%) had positive test results for SARS-CoV-2, and 69 of the 690 nonvaccinated HCWs (10%) also had positive test results for SARS-CoV-2 (data were available for 63 of 69 [91%]). The vaccinated-negative control group consisted of 81 HCWs (Table). The proportion of HCWs who worked in COVID-19 departments was similar among the groups. Symptoms were the common cause for testing among the positive groups (39 of 63 HCWs [62%] in the nonvaccinated-positive group and 17 of 27 [63%] in the vaccinated-positive group), but not in the vaccinated-negative group (18 of 81 HCWs [22%]) (*P* < .001).

**Table.** Characteristics of COVID-19 Vaccinated-Positive Health Care Workers Compared With Nonvaccinated-Positive and Vaccinated-Negative Health Care Workers^a^

Characteristic	No. (%)			*P* value
	Vaccinated-positive (n = 27)	Nonvaccinated-positive (n = 63)	Vaccinated-negative (n = 81)	
Age, mean (SD), y	38 (10)	33 (10)	42 (14)	<.001^b^
Sex				
Female	16 (59)	47 (75)	55 (68)	.33
Male	11 (41)	16 (25)	26 (32)	
Pregnancy	1 (4)	8 (12)	2 (3)	.05
Immunocompromised	3 (11)	1 (2)	3 (4)	.10
Medical sector				
Physician	6 (22)	6 (10)	13 (16)	.50
Nurse	10 (37)	30 (43)	31 (38)	
Worked in a dedicated COVID-19 department	8 (30)	20 (32)	16 (21)	.30
Reason for testing				
Exposure	8 (30)	20 (32)	31 (38)	<.001
Screening	2 (7)	4 (6)	32 (40)	
Symptoms	17 (63)	39 (62)	18 (22)	
Recent exposure^c^				
Household	15 (56)	24 (38)	7 (9)	<.001
No. of positive household members, mean (SD)	2.67 (2.1)	1.7 (1.2)	1.3 (0.7)	.09^d^
At work	2 (7)	17 (27)	26 (31)	.04
Other	2 (7)	4 (6)	1 (1)	.22
Symptoms	22 (81)	56 (89)	18 (22)	.33^e^
Fever	4 (15)	16 (25)	5 (6)	.40^e^
Cough	10 (37)	28 (44)	7 (9)	.64^e^
Coryza	17 (63)	20 (31)	6 (8)	.01^e^
Sore throat	11 (41)	11 (17)	3 (4)	.03^e^
Loss of taste/smell	9 (33)	26 (41)	0	.64^e^
Shortness of breath	3 (11)	10 (16)	3 (4)	.75^e^
ED visit	2 (7)	4 (6)	0	>.99^e^
Hospital admission	1 (4)	2 (3)	0	>.99^e^

Abbreviation: ED, emergency department.

^a^ The vaccinated-positive group consisted of all health care workers who were vaccinated, and had a positive PCR result for SARS-CoV-2 at least 2 weeks after recieving the second dose; nonvaccinated-positive group, all nonvaccinated workers who had a positive result during the same period; and vaccinated-negative group, vaccinated workers who were tested for SARS-CoV-2 and had negative results.

^b^ Analysis of variance.

^c^ Household exposure was defined as dwelling in the same residence with a COVID-19–positive person; at work or other exposures were defined as being exposed for more than 15 minutes to any person with positive test results for SARS-CoV-2 while not wearing proper protective equipment.

^d^ Kruskal-Wallis test for nonparametric data.

^e^ This analysis compared only vaccinated-positive and nonvaccinated-positive health care workers.

Exposure to a positive household member, defined as dwelling in the same residence, was significantly more common among HCWs in the vaccinated-positive group (15 of 27 HWCs [56%]) than in the nonvaccinated-positive group (24 of 63 [38%]) and the vaccinated-negative group (7 of 81 [9%]) (*P* < .001). The OR of exposure to positive household members among vaccinated-positive HCWs was 2.03 (95% CI, 0.74-5.62) compared with nonvaccinated-positive HCWs, and was 12.5 (95% CI, 3.70-43.23) compared with vaccinated-negative HCWs. Albeit not significant, the mean (SD) number of positive household members per case was higher in the vaccinated-positive group at 2.7 (2.1) compared with the nonvaccinated-positive group at 1.7 (1.2) and the vaccinated-negative group at 1.3 (0.7) (*P* = .09). Overall, 1 of 27 HCWs (4%) in the vaccinated-positive group and 2 of 63 (3%) in the nonvaccinated-positive were hospitalized; none died.

## Discussion

This case-control study found that exposure to SARS-CoV-2–positive household members was a risk factor associated with infection among vaccinated HCWs. Household exposure is usually longer and closer than casual exposure or exposure at work and does not include masking or distancing, thus exposing one to a higher infectious dose and being more contagious. Similarly, Kahlert et al^6^ recently found that living with COVID-19–positive household members showed the strongest association with seropositivity among HCWs.

This study had limitations, including the small study size and its retrospective nature. However, we tried to increase the power of the study by selecting 3 negative controls for each case.

The findings of this case-control study suggest reconsideration of quarantining vaccinated people who have had significant exposure to household members who are positive for SARS-CoV-2 infection. This policy has already been implemented successfully in our hospital. Coupled with the current emergence of the SARS-CoV-2 delta variant in Israel and worldwide, our proposal should apply not only to HCWs but to the general population.
